# The strain distribution in the lumbar anterior longitudinal ligament is affected by the loading condition and bony features: An *in vitro* full-field analysis

**DOI:** 10.1371/journal.pone.0227210

**Published:** 2020-01-14

**Authors:** Marco Palanca, Maria Luisa Ruspi, Luca Cristofolini, Christian Liebsch, Tomaso Villa, Marco Brayda-Bruno, Fabio Galbusera, Hans-Joachim Wilke, Luigi La Barbera

**Affiliations:** 1 Department of Industrial Engineering, School of Engineering and Architecture, Alma Mater Studiorum–Università di Bologna, Bologna, Italy; 2 Institute of Orthopaedic Research and Biomechanics, Trauma Research Center Ulm (ZTF), University Hospital Ulm, Ulm, Germany; 3 Laboratory of Biological Structure Mechanics, Department of Chemistry, Materials and Chemical Engineering “G. Natta”, Politecnico di Milano, Milan, Italy; 4 IRCCS Istituto Ortopedico Galeazzi, Milan, Italy; 5 Department of Spine Surgery III, IRCCS Istituto Ortopedico Galeazzi, Milan, Italy; University of Zaragoza, SPAIN

## Abstract

The role of the ligaments is fundamental in determining the spine biomechanics in physiological and pathological conditions. The anterior longitudinal ligament (ALL) is fundamental in constraining motions especially in the sagittal plane. The ALL also confines the intervertebral discs, preventing herniation. The specific contribution of the ALL has indirectly been investigated in the past as a part of whole spine segments where the structural flexibility was measured. The mechanical properties of isolated ALL have been measured as well. The strain distribution in the ALL has never been measured under pseudo-physiological conditions, as part of multi-vertebra spine segments. This would help elucidate the biomechanical function of the ALL. The aim of this study was to investigate in depth the biomechanical function of the ALL in front of the lumbar vertebrae and of the intervertebral disc. Five lumbar cadaveric spine specimens were subjected to different loading scenarios (flexion-extension, lateral bending, axial torsion) using a state-of-the-art spine tester. The full-field strain distribution on the anterior surface was measured using digital image correlation (DIC) adapted and validated for application to spine segments. The measured strain maps were highly inhomogeneous: the ALL was generally more strained in front of the discs than in front of the vertebrae, with some locally higher strains both imputable to ligament fibers and related to local bony defects. The strain distributions were significantly different among the loading configurations, but also between opposite directions of loading (flexion vs. extension, right vs. left lateral bending, clockwise vs. counterclockwise torsion). This study allowed for the first time to assess the biomechanical behaviour of the anterior longitudinal ligament for the different loading of the spine. We were able to identify both the average trends, and the local effects related to osteophytes, a key feature indicative of spine degeneration.

## Introduction

The anterior longitudinal ligament (ALL) is a fundamental component of the spine. It covers the anterior aspect of the spine running along the entire length of the spine [1]. To elucidate the contribution of the different anatomical elements to the biomechanics of the spine, it is important to identify the specific behaviour of the ALL. Microdissection and anatomical studies showed that the ALL comprises distinct layers. More superficial fibers attach to central regions of the vertebrae, running longitudinally and spanning up to 4–5 functional spinal units (FSUs—consisting of two vertebrae and one intervertebral disc). Much shorter intermediate fibers cover more intervertebral discs (IVDs) and insert onto the anterior aspect of the adjacent vertebrae, spanning 2–3 FSUs. The deepest layer covers longitudinally and obliquely (i.e.: alar fibers) a single IVD [2]. The ALL deeper fibers are solidly attached on the periosteum of the vertebrae and they continue in the external lamellae of the anterior part of the IVD [3]. The ALL has an important role in stabilizing and limiting movements in the sagittal plane, and in confining the anterior wall of the intervertebral discs (IVD). Its mechanical role has direct implications on low-back pain, since it limits primary and coupled motions in extension. As the ALL can prevent the bulging of the IVD, it contributes to maintain the height between two adjacent vertebrae in flexion. Consequently, the ALL prevents closure of the foraminal spaces and compression of the nerve roots. Such effects are even more important in case of disc degeneration. Furthermore, the ALL, like most spine ligaments, is rich with mechanoreceptors and plays a fundamental role in the neuromotor control [4].

The biomechanical function and strain distribution in the vertebrae and IVD have been investigated *in vitro* in detail [5–7]. Often, only FSUs were tested [7,8] whereas multi-vertebra spine specimens should be preferred in *in vitro* tests [9] for the investigation of those ligaments spanning more than one FSU, indeed this represents a more realistic and complete loading condition. From these tests, the range of motion [10–12] and/or the neutral zone and stiffness [13–15] under the different physiological loading conditions were evaluated for the different spinal levels. This type of measurements provides useful information about the global description of the spine biomechanics, but it is unable to elucidate in detail what happens locally on the spine segment.

The investigations on the spinal ligaments are somehow limited. Specifically, the ALL, which is one of the strongest ligamentous structure in the spine, has only partially been investigated so far. Generally, the ALL was tested separately at the tissue level: evaluating the mechanical properties of dissected tissue specimens [16–18], and at the system level: evaluating its structural behaviour when it was included in spine tests [9,19–23]. However, a biomechanical characterization of the ALL tissue when it was incorporated in the spine, with the typical and complex loading conditions, is missing. No studies were found in which the strain distribution was measured on the ALL, in its complete mechanical and anatomical complex, as part of a multi-vertebra spinal segments, and under different loading conditions representative of physiological loading.

In this work, the evaluation of multi-vertebra spine segments (i.e.: 7/8 vertebrae and 6/7 intervertebral discs) through flexibility tests was integrated with a full-field measurement of the strain distribution of the anterior surface [24–26]. The overall aim of this study was to investigate in depth the biomechanical function of the ALL in front of the lumbar vertebrae (L3-L5) and of the intervertebral discs.

Specifically, we aimed measuring the strain distribution in the ALL for different directions of motions under pure moments, and so understanding how the strain distribution changes through the progression of the loading cycle analyzing discrete steps.

We hypothesized the ALL undergoes non-uniform strain distribution when the spine segment is subjected to pure moments, potentially related to the unique specimens’ anatomy/morphology (e.g.: presence of osteophytes); moreover, opposite loading directions (e.g.: flexion/extension, or right/left lateral bending, or clockwise/counterclockwise axial torsion) translate to non-mirrored strain distributions.

## Materials and methods

### Study design

This study was approved by the Institutional Review Board (Ethikkommission) of Ulm University, (Document of approval Nr. 307/17). In order to investigate the strain distribution on the anterior longitudinal ligament in the lumbar region, cadaveric multi-vertebra spinal segments were subjected to non-destructive pure moments in different directions, with a state-of-the-art spine tester. The tests were performed twice on each specimen under identical conditions. Preliminarily, the range of motion was measured, with an optical motion tracking system, to allow comparisons of the tests results. Subsequently, the strain distributions on the anterior surface of the L3-L5 region, were measured by means of digital image correlation (DIC) with a recently validated protocol, for identifying stretched/compressed regions. The strain distributions were analyzed firstly qualitatively, and then quantitatively.

### Specimens

Five fresh-frozen human thoracolumbar spine segments were obtained through an ethically-approved international donation program (Science Care Inc., Phoenix, AZ). The donors were all Caucasian, three males and two females (Table 1). Inclusion criteria were: no history of spine fracture or major spine deformity; no tumour; physically active, i.e.: ambulatory activities and daily living activities, up to date of death. The median age of the subjects at the time of death was 62 years and their median weight was 133 kg. To check the state of degeneration and determine the bone mineral density (BMD), each specimen was scanned using a calibrated clinical computed tomography (CT) scanner (Philips Brilliance 64, Philips Healthcare, Cleveland, USA). The BMD was expressed averaging the measurements collected on the trabecular bone of L1, L2 and L3 vertebrae.

**Table 1:** Details of the specimens: the first columns report the donor’s information. The last three columns report the radiographic assessment for L4 and L5, evaluated through CT scans according to [27] (the CT scans for each individual specimen is reported in the S1 Fig). The IVD height loss and osteophytes formations were assessed according to [27]. Independently of the specimen extension T11-S1 or T12-S1, in all specimens the region of interest was the L3-L5 segment.

Specimen number	Segment	Gender	Age at death (years)	Height (cm)	Weight (kg)	BMD (mg/cm^3^)	Disc height loss	Osteophytes formations	Overall degree of degeneration—Other remarks
#1	T11-S1	M	60	N.A.	N.A.	153	No remarks (grade 0)	No osteophytes (grade 0)	Healthy
#2	T11-S1	M	66	183	141	82	Mild (25%, grade 1)	Moderate (grade 2)	Mild degenerative signs (grade 1)
#3	T11-S1	M	62	178	164	94	Moderate (42%, grade 2)	Moderate (grade 2)	Moderate degenerative signs (grade 2)—thickening of L4 anterior cortical wall.
#4	T11-S1	F	60	163	114	123	Moderate (40%, grade 2); T12-L1 and L4-L5 discs herniated towards L1 and L5, resp.	Moderate (grade 2)	Moderate degenerative signs (grade 2)—mild scoliosis (Cobb angle T12-L5 of 10°); concave L5 superior endplate
#5	T12-S1	F	63	157	125	157	Moderate (33%, grade 2)	Severe (grade 3)	Moderate degenerate signs (grade 2)
**Median**	**-**	**-**	**62**	**170**	**133**	**123**	**-**	**-**	**-**

The volumetric CT reconstructions of the specimens are available in the S1 Fig, otherwise a conventional x-rays image (Faxitron 43805N, Hewlett Packard, Palo Alto, USA) is provided. No critical damages were observed; however, most specimens showed some osteophytes as can be expected with elderly donors. The osteophytes and reduction of IVD height was quantified with objective metrics, according to [27].

The soft tissues (muscles, fat) on the anterior side of the spines were carefully removed to expose the anterior longitudinal ligament and the lateral side of the vertebral bone and of the intervertebral discs; the posterior elements were left in place [28]. The upper half of the most cranial vertebra and the lower half of the most caudal vertebra were embedded in poly-methyl-methacrylate (PMMA, Technovit 3040, Heraeus Kulzer, Werheim, Germany) blocks. After the preparation, the specimens were frozen in plastic bags at -20°C until the day of the tests. Thawing at 6°C for 10 h prior to preparation and testing of the specimens were performed within 20 h to avoid alteration of their mechanical properties.

### Mechanical loading

**Fig 1:**
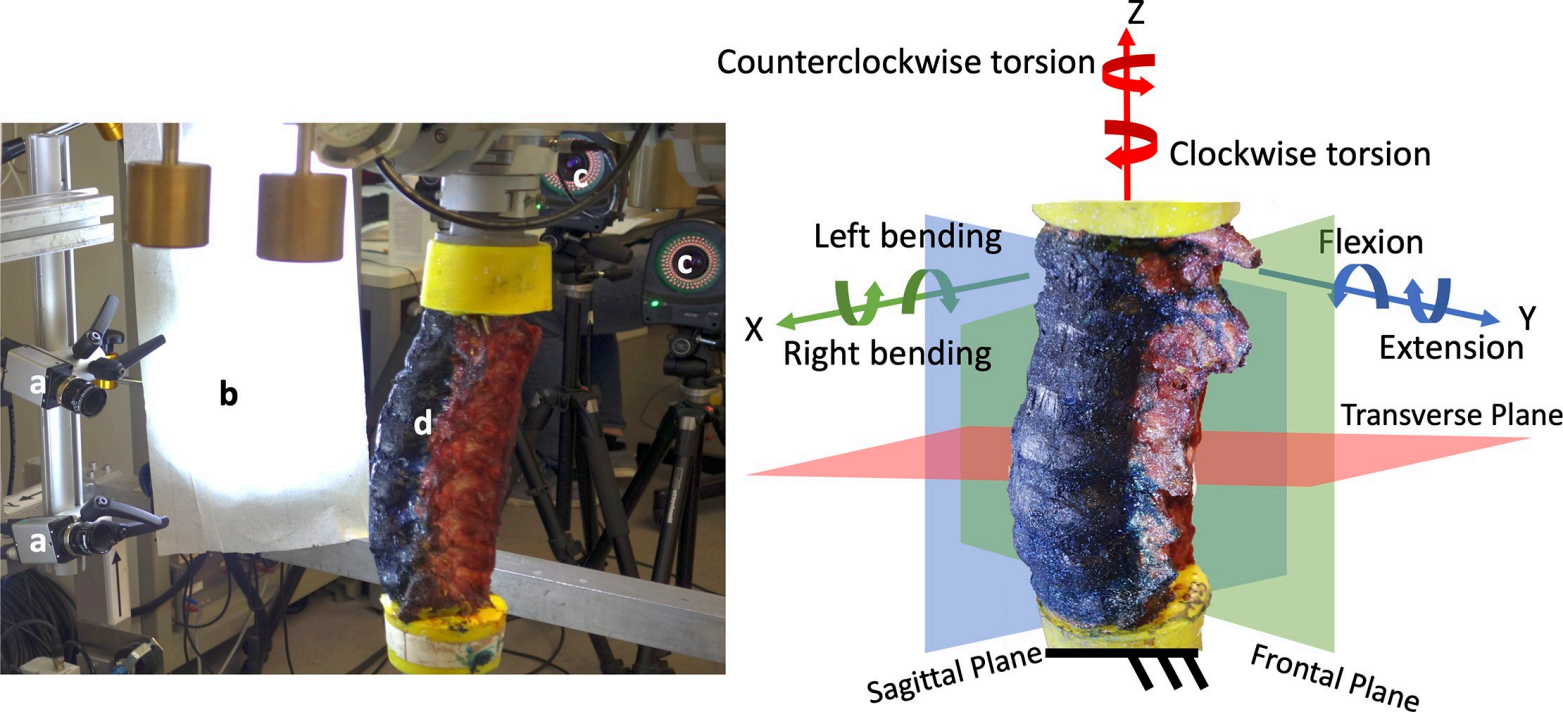
Left: overview of the testing setup showing (a) the DIC cameras, (b) the DIC light system, (c) the optoelectronic cameras and (d) the specimen. Right: detail of the spine segment. The white-on-black pattern is visible. Also shown is the three-dimensional coordinate system used by the different measurement tools: the transverse plane of the spine segment corresponds to the xy-plane of the coordinate system, the sagittal plane to the xz-plane and the frontal plane to the yz-plane. The X-axis is forward, the Y-axis left and the Z-axis cranial.

All specimens were tested at room temperature (ca. 23°C) and the hydration was preserved spraying saline solution during the tests. In order to mount the specimens in a universal spine tester, flanges were fixed to the PMMA blocks [28,29] so that the L3-L5 segment was vertical. Initially the cranial side was connected to the top of the spine simulator with the gimbal with three integrated stepper motors (FT 1500/40, Schunk GmbH & Co. KG, Lauffen/Neckar, Germany), then the caudal side with the natural slope for each specimen was fixed on the bottom side of the testing machine (Fig 1).

The coordinate system of [28] was used in this work. Each specimen was tested without any preload in flexion/extension (My), right/left lateral bending (Mx) and clockwise/counterclockwise axial torsion (Mz) applying pure moments (M) up to +/-7.5 Nm. Flexion/extension and lateral bending were applied at a rate of 1°/s, while the axial torsion was applied at a rate of 0.5°/s. As the thoraco-lumbar spine is about twice as stiff in torsion, the rate in torsion was half of that in bending, so as to reach the fully loaded condition in approximately the same time. Each test consisted of three consecutive cycles for each direction of loading: the first two cycles for pre-conditioning, the last one for the actual analysis [30]. All motions started and finished in the unloaded neutral position. In order to avoid application of any additional undesired loading component, the specimens were unconstrained in the other uncontrolled five degrees of freedom. A six-components load cell (FT 1500/40, Schunk GmbH & Co. KG, Lauffen/Neckar, Germany) measured the moments and the forces applied.

### Measurement of structural properties

In order to confirm that the overall kinematics of the specimens was consistent with the literature, the mechanical tests were first performed with a motion tracking system. Each single vertebra was equipped with three spherical reflective markers, which were attached frontally and laterally to the vertebral body. The motion of the single vertebrae was simultaneously captured with six cameras of the optical system (Mod. MX13, Vicon Motion Systems Ltd., Oxford, UK), synchronized with the mechanical loading apparatus. After evaluating the relative intervertebral motions using the software Nexus 1.8.5 (Vicon Motion Systems Ltd.), the kinematic data were matched with the moment data to analyze the resulting load-deformation curves. The global range of motion (ROM) as well as the global neutral zone (NZ) of each motion segment between T12 and the sacrum were quantified using dedicated scripts (in MatLab R2014b, MathWorks, Natick, USA).

### Measurement of the local distribution of the strains

In order to measure the full-field strain distribution on the anterior spinal ligaments, the same mechanical tests were performed while a 3D DIC system was used. A white-on-black speckle pattern was prepared before the test on the anterior surface of the specimens following an optimized procedure [25]. The dark background was prepared staining the ALL, the intervertebral discs and the vertebrae with a solution of methylene-blue (4 g of methylene-blue per 100 ml of water) until a uniform dark background was obtained. The white dots were created with a white water-based paint (Q250201 Bianco Opaco, Chrèon, Italy) diluted at 40% with water and sprayed with an airbrush-airgun (AZ3-THE-2, nozzle 1.8 mm, Antes-Iwata, Italy) with 100kPa air pressure, from a distance of 300 mm. Such settings were refined in order to obtain the optimal size of the speckle dots [31] following a validated protocol [32,33].

The DIC system (Q400, Dantec Dynamics, Denmark) was configured with two 5 Mpixels cameras (2440x2050, 8-bit, black-and-white) equipped with high-quality metrology-standard 17 mm lens (Xenoplan, Schneider-Kreuznach, Germany; 65 mm equivalent) to acquire images of the specimens providing a stereoscopic vision. A directional custom system of LEDs (10’000 lumen in total) was placed to light up the specimen with oblique light minimizing the glares on the specimen typical of direct illumination. The cameras were placed at a distance of 540 mm from the specimen. The cameras were aligned vertically in order to take advantage of the sensor shape in framing the region of interest (ROI) of the spine segment (three vertebrae and two intervertebral discs–from L3 to L5) without scarifying the measurement spatial resolution, Fig 1). In this configuration, the field of view was of about 120 mm by 160 mm, it was depending on the individual specimen, resulting in a pixel size of about 0.08 mm, and a depth of field of 70 mm with the aperture adopted (f/22). Images of the ROI were acquired at 5 frames per second.

To enable the stereoscopic reconstruction within the measurement volume and correct the distortion of the lenses, a calibration was performed before each acquisition using a proprietary calibration target (Al4-BMB-9x9, Dantec Dynamics). The analysis of displacements and strains through correlation of the images was achieved using the proprietary software Istra 4D (v4.3.1, Dantec Dynamics, Denmark). The maximum (eps_1_) and minimum (eps_2_) engineering principal strains, as well as their direction, were computed using a facet size between 39 and 59 pixels, a grid spacing between the facets of 4 pixels and contour smoothing with a kernel size of 5x5 facets.

### Measurement uncertainties, Metrics and statistical analysis

#### Uncertainties

The accuracy of the motion tracking system was evaluated using special custom-made calibration object.

An extensive validation and optimization of the DIC measurement system and protocols were previously performed [25,33]. Here, an estimation of the unavoidable measurement uncertainties was performed just before each mechanical test. A couple of images of each specimen in the unloaded condition (zero-strain) was acquired. The images were analyzed using the chosen settings. The systematic and random errors were evaluated as the mean and standard deviation of eps_1_ and eps_2_ over the entire ROI, which theoretically should be zero.

#### Metrics and statistics

Global (T12-S1) range of motion (ROM) was defined as the maximum deflection of the respective motion segment at full load (7.5 Nm). The global neutral zone (NZ) was evaluated as the difference of the angle at 0 Nm of the hysteresis cycle. The NZ specifies the motion of the specimen in the unloaded region, representing the laxity [28].

The full-field eps_1_ and eps_2_ maps were computed by the DIC system during the entire load cycle (https://doi.org/10.6084/m9.figshare.11397951). For a qualitative analysis, the strain maps, in the region of interest from L3 to L5 were reported for each loading scenario during the progression of the load.

For a quantitative analysis, two sub-regions of interest (sub-ROIs): in front of the L4 vertebra and in front of the L4-L5 IVD, were defined. For each sub-ROIs, the strain field at the maximum load was analyzed through a MatLab script computing the principal strains medians. To assess the significance of the difference between the strains in front of the vertebra and in front of the IVD, the medians over such areas were compared with the two-sample Mann-Whitney test for each loading scenario. To describe how the principal strains were distributed in the circumferential direction of the ALL in front of the L4 vertebra and in front of the L4-L5 IVD, the median over cranio-caudal (vertical) lines were computed, separately, over the vertebra and over the IVD, both for eps_1_ and eps_2_, for each specimen. Similarly, to describe how the principal strains were distributed in the cranio-caudal direction of the ALL in front of the vertebra and in front of the IVD, the median over circumferential (horizontal) lines were computed, separately, over the vertebra and over the IVDs. Then, the data from the five specimens were pooled and the median trend plotted together with the standard deviation. As the sub-ROIs were dimensionally different in the different specimens and the number of measurement points is connected with the physical dimension of the spine segments, the data were re-sampled over the same number of points. In order to assess the significance of alterations of such distribution of strain in relation to the different loading scenarios, a two-sample Kolmogorov-Smirnov test was applied both to the circumferential and cranio-caudal strain distributions of the L4 vertebra and the caudal IVD, discriminating the opposite directions of loading.

## Results

All the tests were successfully performed with no visible damage of the specimens. A preliminary check of the bending moment-rotation plots from the spine tester confirmed that the difference between the two series of loading cycles (i.e.: those to measure the structural properties with the motion tracking system, and those to measure the strain distribution with the DIC) were smaller than 5° with a rotation of 33° at full load. The only problems encountered was the poor correlation for flexion-extension for specimen #1 and the loss of the dataset of specimen #4 for extension.

### Measurement uncertainties

An in-house validation showed that the motion tracking system has an accuracy of better than 0.1 mm and better than 0.1°. The zero-strain tests, before each test, indicated that DIC-measured strains had a systematic error lower than 20 microstrain and a random error lower than 60 microstrain.

### Structural properties

The median global ROM between T12 and the sacrum at 7.5 Nm was 12.0° in flexion-extension (range: 9.7°–14.6°), 13.6° (range: 12.4°–24.9°) in lateral bending and 7.8° in axial torsion (range: 4.6°–9.3°). The global NZ between T12 and the sacrum was 3.7° in flexion-extension (range: 1.9°–5.9°), 6.2° in lateral bending (range: 4.4°–20.1°) and 0.8° in axial torsion (range: 0.2°–1.8°).

### Local distribution of strains

**Fig 2:**
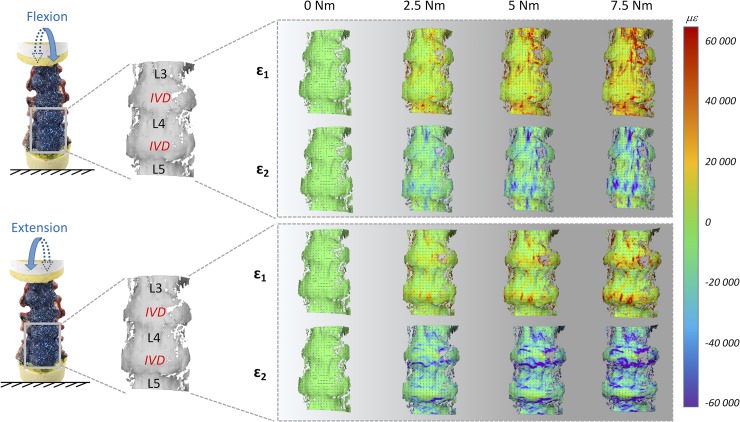
Flexion and extension: Maximum (eps_1_) and minimum (eps _2_) principal strain fields during the progression of the loading cycle (0.0, 2.5, 5.0, 7.5 Nm) in both opposite directions. The images on the left show the actual specimen under load and the correlated (the vertebrae and IVDs are labelled). The false-colours maps show the non-uniform distribution of strain. The black dashes indicate the principal strain directions. A typical specimen (#2) is shown here. Similar patterns were observed in all 5 specimens (the strain maps for all the individual specimens are reported in the S1 Fig, S1 and S2 Dataset). A movie of the entire loading cycle is available on https://doi.org/10.6084/m9.figshare.11397951.

**Fig 3:**
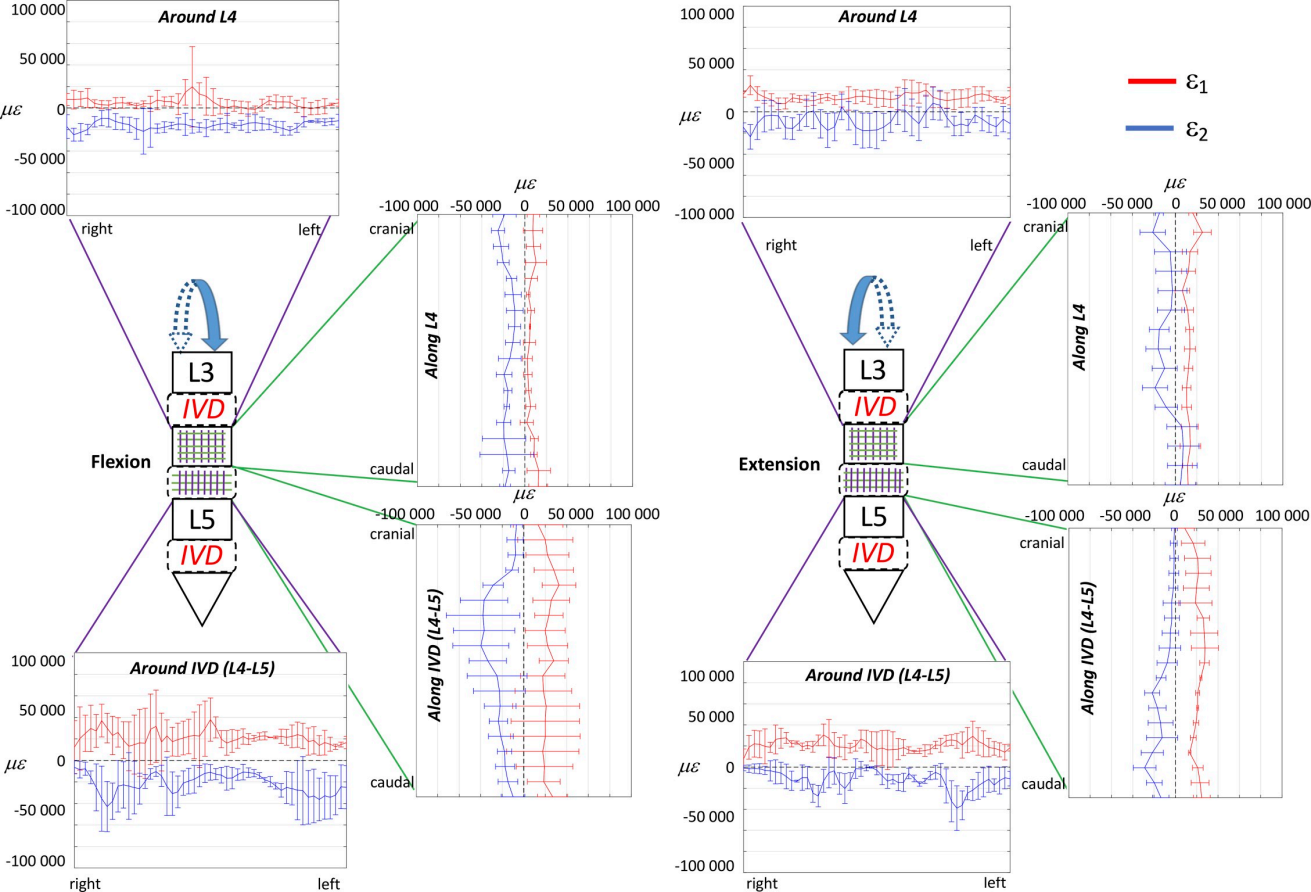
Flexion and extension at the maximum loading (7.5 Nm): to describe the strain distribution of the ALL in the circumferential direction, the median over cranio-caudal lines were computed in the sub-ROI in front of the L4 vertebra and in the sub-ROI in front of the L4-L5 IVD. Similarly, to describe the strain distribution of the ALL in the cranio-caudal direction, the median over circumferential lines were computed in the sub-ROI in front of the L4 vertebra and in the sub-ROI in front of the L4-L5 IVD. The plots show the distribution ofeps_1_ (red) and eps _2_ (blue) circumferentially the L4 and the IVD (purple lines) and in the cranio-caudal direction of L4 and the IVD (green lines). The median and standard deviation within the five specimens are reported.

**Fig 4:**
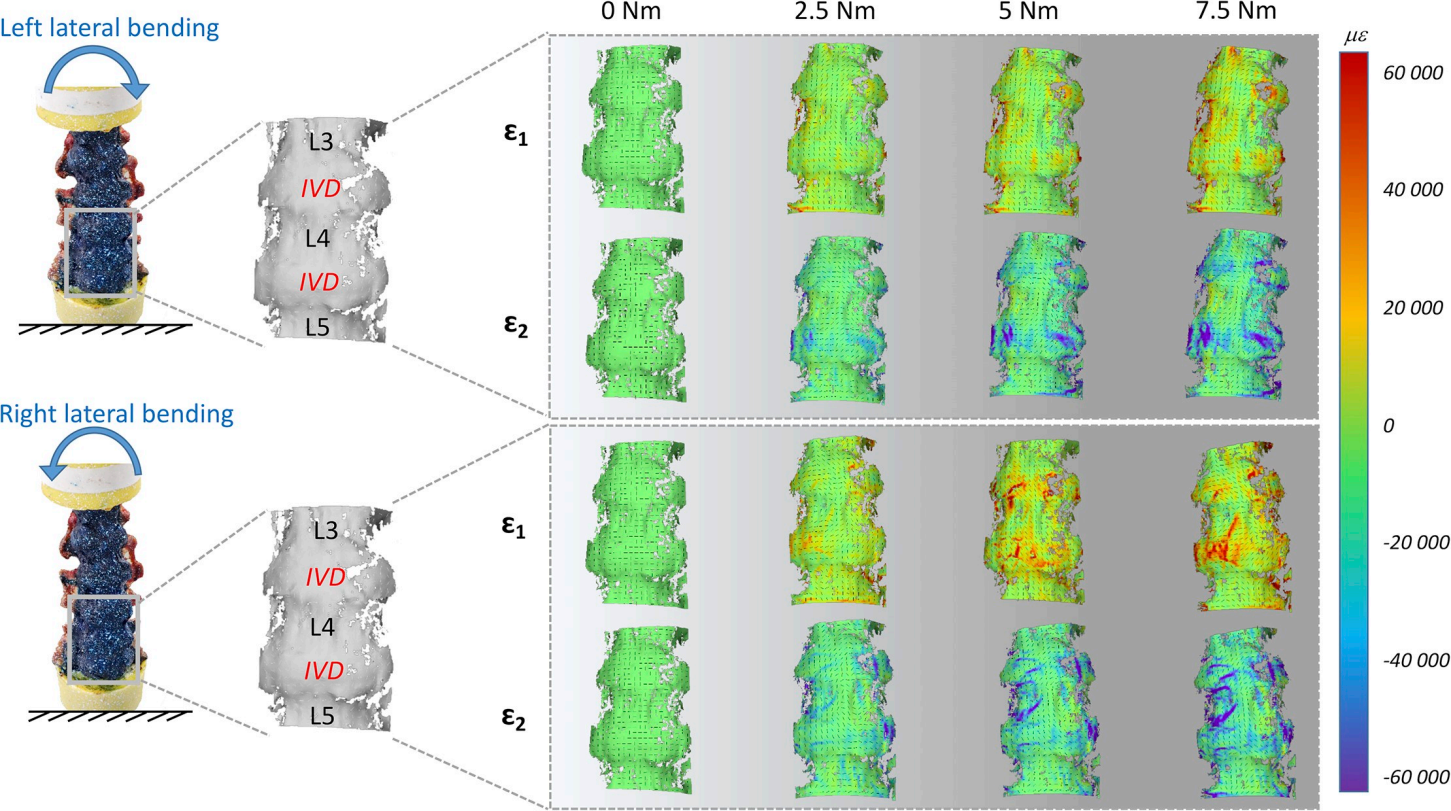
Lateral bending: Maximum (eps _1_) and minimum (eps _2_) principal strain fields during the progression of the loading cycle (0.0, 2.5, 5.0, 7.5 Nm) in both opposite directions. The images on the left show the actual specimen under load and the correlated (the vertebrae and IVDs are labelled). The false-colours maps show the non-uniform distribution of strain. The black dashes indicate the principal strain directions. A typical specimen (#2) is shown here. Similar patterns were observed in all 5 specimens (the strain maps for all the individual specimens are reported in the S1 Fig, S1 and S2 Dataset). A movie of the entire loading cycle is available on https://doi.org/10.6084/m9.figshare.11397951.

**Fig 5:**
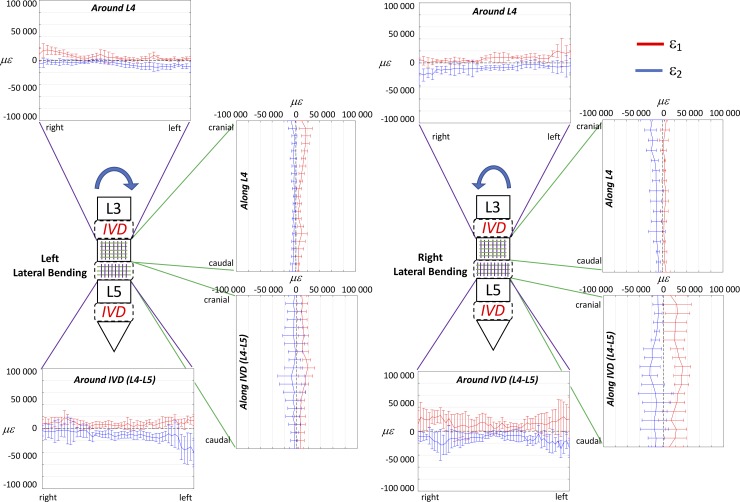
Lateral bending at the maximum loading (7.5 Nm): to describe the strain distribution of the ALL in the circumferential direction L, the median over cranio-caudal lines were computed in the sub-ROI in front of the L4 vertebra and in the sub-ROI in front of the L4-L5 IVD. Similarly, to describe the strain distribution of the ALL in the cranio-caudal direction, the median over circumferential lines were computed in the sub-ROI in front of the L4 vertebra and in the sub-ROI in front of the L4-L5 IVD. The plots show the distribution of eps _1_ (red) and eps _2_ (blue) circumferentially the L4 and the IVD (purple lines) and in the cranio-caudal direction of the L4 and the IVD (green lines). The median and standard deviation within the five specimens are reported.

**Fig 6:**
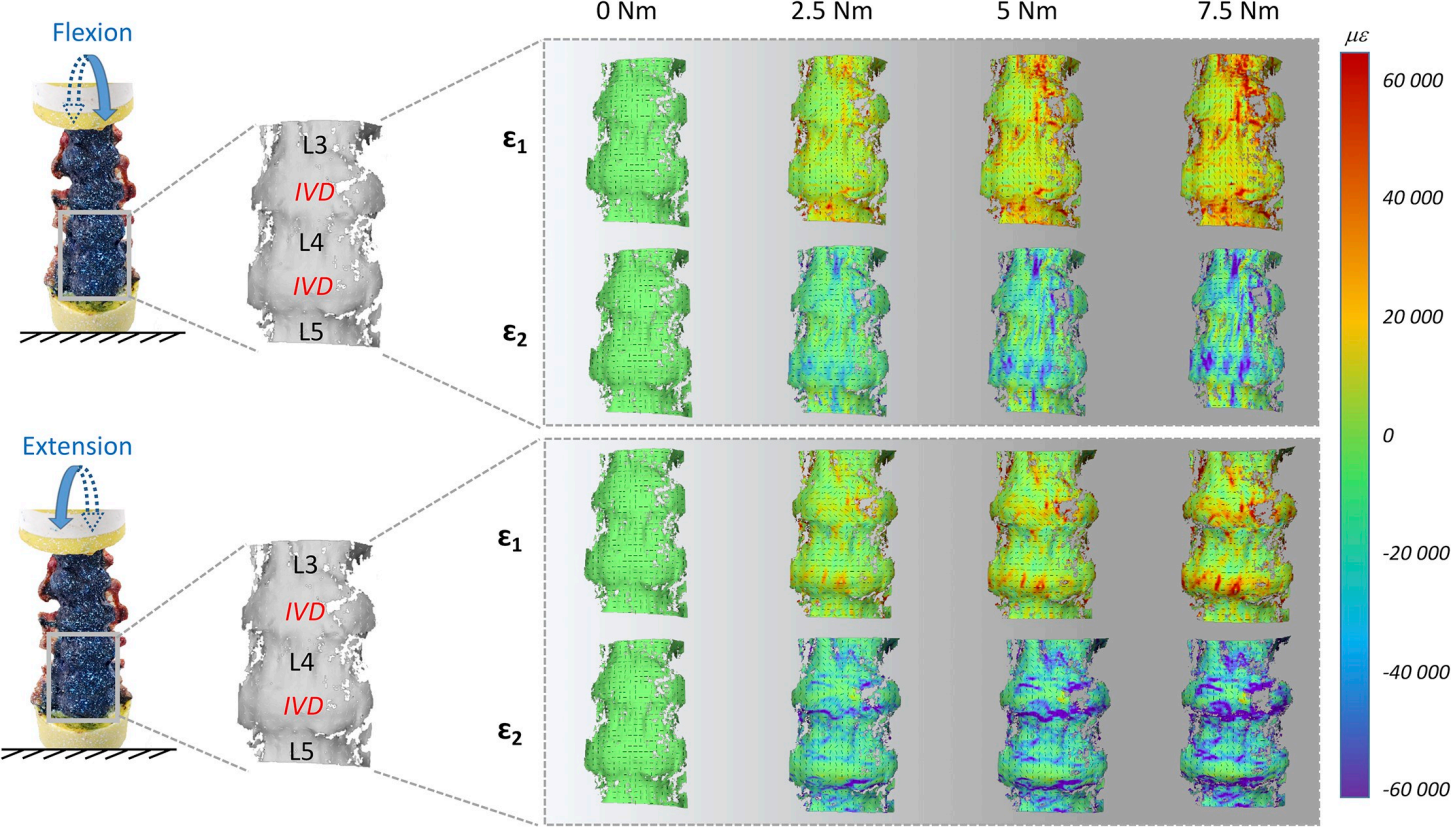
Axial Torsion: Maximum (eps _1_) and minimum (eps _2_) principal strain fields during the progression of the loading cycle (0.0, 2.5, 5.0, 7.5 Nm) in both opposite directions. The images on the left show the actual specimen under load and the correlated (the vertebrae and IVDs are labelled). The false-colours maps show the non-uniform distribution of strain. The black dashes indicate the principal strain directions. A typical specimen (#2) is shown here. Similar patterns were observed in all 5 specimens (the strain maps for all the individual specimens are reported in the S1 Fig, S1 and S2 Dataset). A movie of the entire loading cycle is available on https://doi.org/10.6084/m9.figshare.11397951.

**Fig 7:**
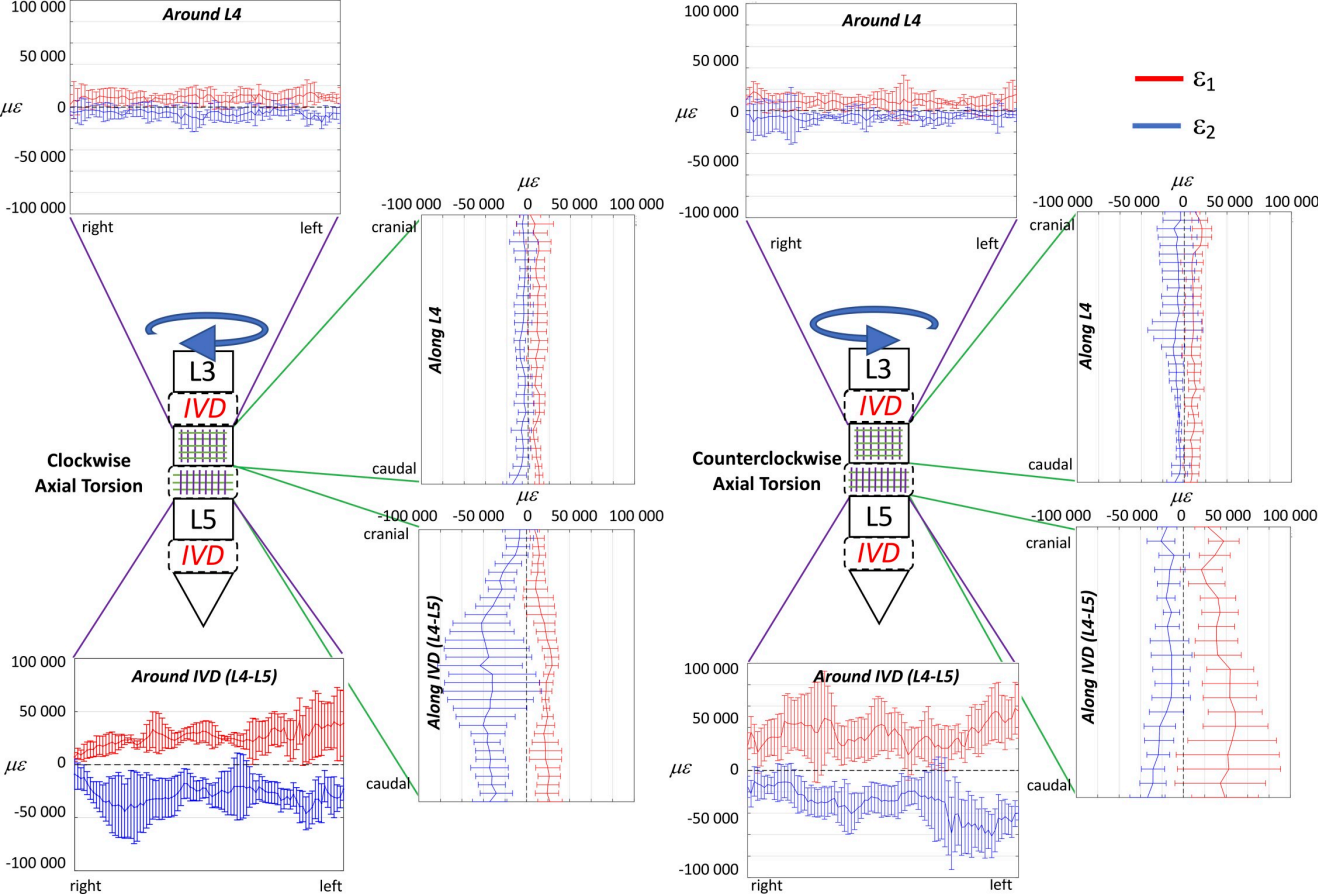
Axial Torsion at the maximum loading (7.5 Nm): to describe the strain distribution of the ALL in the circumferential direction, the median over cranio-caudal lines were computed in the sub-ROI in front of the L4 vertebra and in the sub-ROI in front of the L4-L5 IVD. Similarly, to describe the strain distribution of the ALL in the cranio-caudal direction, the median over circumferential lines were computed in the sub-ROI in front of the L4 vertebra and in the sub-ROI in front of the L4-L5 IVD. The plots show the distribution of eps _1_ (red) and eps _2_ (blue) circumferential direction of the L4 and the IVD (purple lines) and in the cranio-caudal direction of the L4 and the IVD (green lines). The median and standard deviation within the five specimens are reported.

The full-field strain maps showed a non-homogeneous distribution in the ALL (Figs 2–7). The peak values of the maximum and minimum principal strains had the same order of magnitude, in all loading scenarios. The different loading scenarios generated different strain patterns. These strain maps allowed to identify which portions of the ALL were actually working, in tension or compression, and which portions were unstrained (Figs 3, 5 and 7). Some stripes characterized by larger strains were visible in all specimens with a preferential cranio-caudal orientation (Figs 2, 4 and 6). In most specimens, also some spots with larger strains were visible, especially close to the endplates.

**Fig 8:**
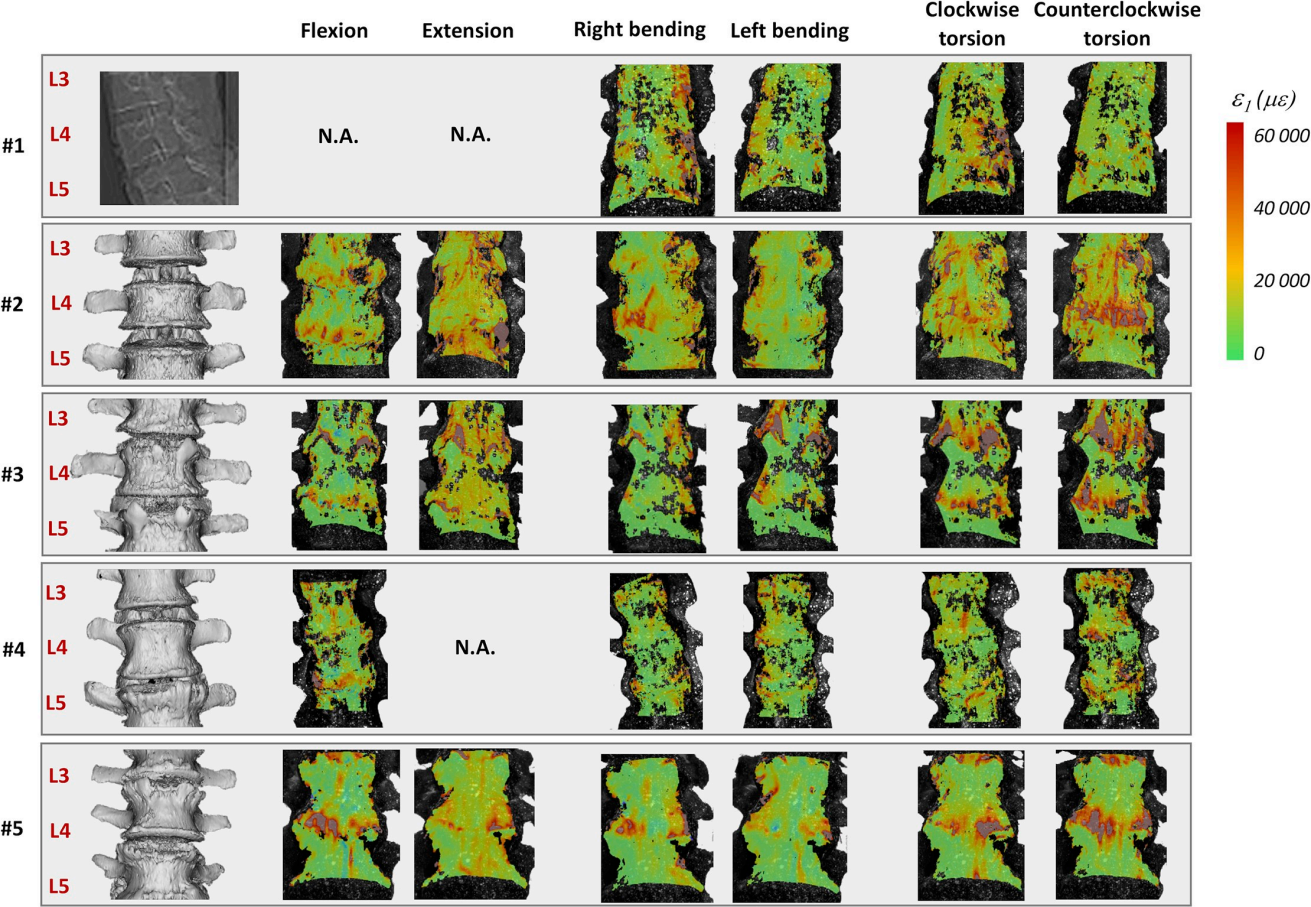
Specimen-specific analysis of the strain distribution: the CT images of each specimen are reported on the left (for Specimen #1 the CT was not available). On the right, the distribution of the maximum principal strain (eps_1_) are plotted for each loading condition, at full load (7.5 Nm). The minimum principal strain (eps_2_) and the analysis of the circumferential and cranio-caudal distribution of strain of the vertebra and the IVD are reported in the S1 Fig, S1 and S2 Dataset.

While common trends were visible in all specimens, inter-specimen differences were found in association with specific bony-defects and individual defects highlighted by the CT images (Fig 8).

#### General trends for flexion/extension

During the application of flexion/extension, strains increased more pronouncedly, in absolute value, in the ALL in the areas in front of the IVDs, and especially close to the endplates (Fig 2). The same regions reached the maximum strain values when the full load was applied. At the maximum flexion, the median eps_1_ and eps_2_ over the portion of the ALL in front of the L4 vertebra were respectively 3910 microstrain and -15170 microstrain (median between 5 specimens); in front of the IVDs eps_1_ and eps_2_ were respectively 19160 and -23020 microstrain. At the maximum extension, the median eps_1_ and eps_2_ in the ALL in front of L4 were respectively 13890 microstrain and -1890 microstrain (median between 5 specimens); in front of the IVDs eps_1_ and eps_2_ were respectively 18730 and -10710 microstrain. The only significant difference was found for flexion between eps_1_ in front of the IVDs and in front of the L4 vertebra (two-sample Mann-Whitney, Table 2). The median values for the individual specimens are reported in the S1 Fig.

**Table 2:** The reported p-values show the statistical significance of the difference between the median on the vertebrae and intervertebral discs for the same loading condition (two-sample Mann-Whitney test). The median of strains on the ALL in front of the L4 vertebra and the L4-L5 intervertebral disc were examined.

	Maximum princ strain (eps_1_)	Minimum princ strain (eps_2_)
	Vertebra Vs IVD	Vertebra Vs IVD
Flexion	p = 0.03 (*)	p = 0.34
Extension	p = 0.10	p = 0.70
Left Bending	p = 0.10	p = 0.42
Right Bending	p = 0.06	p = 0.31
Clockwise torsion	p = 0.03 (*)	p = 0.01 (*)
Counterclockwise torsion	p = 0.03 (*)	p = 0.02 (*)

Note

(*) highlights significant differences (p<0.05).

During flexion, eps_1_ were circumferential, indicating an axial compression. During extension eps_1_ were directed longitudinally, indicating traction of the ALL. The direction of principal strains in the ALL did not change during the progression of the load.

The plot showing the distribution of eps_1_ and eps_2_ in the cranio-caudal and circumferential directions of the ALL highlighted larger strain in front of the discs with respect to the vertebrae, both in flexion and extension (Fig 3). While in extension, the strains were quite uniformly distributed both in the circumferential and in cranio-caudal direction of the vertebra and disc, in flexion some regions were more strained: the intervertebral disc, at mid-height, and at its right and left extremities. The distributions of strains were significantly different between flexion and extension both in the circumferential and in cranio-caudal direction of the ALL, for eps_1_ only in front of the vertebra, and for eps_2_ both in front of the vertebra and the IVD (two-sample Kolmogorov-Smirnov, Table 3).

**Table 3:** The reported p-values show the statistical significance of the difference between the trends for opposite directions of loading (two-sample Kolmogorov-Smirnov). The distribution of strains (Figs 5, 6 and 7) in the cranio-caudal direction of the L4 vertebra and the L4-L5 intervertebral disc (computed as median strains over circumferential lines), and the distribution of strains in the circumferential direction of the L4 vertebra and the L4-L5 intervertebral disc (computed as median strains over cranio-caudal lines) were examined for the different loading scenarios.

	Maximum princ strain (eps_1_)		Minimum princ strain (eps_2_)
Position, direction	**Flexion Vs Extension**		
L4, cranio-caudal	p = 8.9 x 10^−6^ (*)		p = 2.8 x 10^−3^ (*)
IVD, cranio-caudal	p = 7.4 x 10^−1^		p = 1.8 x 10^−2^ (*)
L4, circumferential	p = 1.3 x 10^−13^ (*)		p = 8.2 x 10^−7^ (*)
IVD, circumferential	p = 5.2 x 10^−1^		p = 3.5 x 10^−5^ (*)
	**Right Vs Left** **Lateral Bending**		
L4, cranio-caudal	p = 1.5 x 10^−4^ (*)		p = 2.4 x 10^−8^ (*)
IVD, cranio-caudal	p = 5.3 x 10^−6^ (*)		p = 1.6 x 10^−9^ (*)
L4, circumferential	p = 1.5 x 10^−1^		p = 2.8 x 10^−2^ (*)
IVD, circumferential	p = 1.4 x 10^−3^ (*)		p = 8.2 x 10^−2^
	**Clockwise Vs Counterclockwise** **Axial Torsion**		
L4, cranio-caudal	p = 8.2 x 10^−1^		p = 6.8 x 10^−3^ (*)
IVD, cranio-caudal	p = 4.2 x 10^−9^ (*)		p = 2.4 x 10^−5^ (*)
L4, circumferential	p = 1.5 x 10^−1^		p = 3.7 x 10^−1^
IVD, circumferential	p = 1.9 x 10^−8^ (*)		p = 3.9 x 10^−2^ (*)

Note

(*) highlights significant differences (p<0.05)

#### General trends for lateral bending

During the application of lateral bending (Fig 4), strains markedly increased in regions both in front of the IVDs and in front of the vertebra. Those regions initially more strained, also reached the maximum strain values at full load. The median eps_1_ and eps_2_ in front of the vertebra, at the maximum left bending, were respectively 4250 microstrain and -6600 microstrain; in front of the IVDs were 8510 microstrain and -10090 microstrain. The median eps_1_ and eps_2_ in front of the vertebra, at the maximum right bending, were respectively 6363 microstrain and -9570 microstrain; in front of the IVDs were respectively 15590 microstrain and -11030 microstrain. None of these differences between the vertebra and the IVD was statistically significant (two-sample Mann-Whitney, Table 2). The median values for the individual specimens are reported in the S1 Fig. The eps_1_ had circumferential direction in the compressed side (left side for the left lateral bending, and vice versa) and longitudinal direction in the tensile side (right side for the left lateral bending, and vice versa).

The trends of strain circumferentially the spine segment for the right and left lateral bending were mirrored with respect to the vertical axis (Fig 5). However, there were differences in magnitude, with larger strains for the right lateral bending, compared to left. No large differences between the two lateral bending scenarios were found in the cranio-caudal strain distribution of the spine segment. The distribution of strains over the vertebra L4 were significantly different between right and left bending, with the exception of eps_1_ circumferentially the vertebra; the distribution over the IVD were significantly different, with the exception of eps_2_ circumferentially the IVD (two-sample Kolmogorov-Smirnov, Table 3).

#### General trends for axial torsion

During the application of torsion (Fig 6), the strains had a visible twisting trend and increased more pronouncedly in front of the IVD. The median eps_1_ and eps_2_, at the maximum clockwise torsion, in front of the vertebra were respectively 7720 microstrain and -5180 microstrain; in front of the IVDs were respectively 23170 microstrain and -23420 microstrain. The median eps_1_ and eps_2_, at the maximum counterclockwise torsion, in front of the vertebrae were respectively 7350 microstrain and -3860 microstrain; in front of the IVDs were 38880 microstrain and -31340 microstrain. All these differences between the vertebra and the IVD were statistically significant (two-sample Mann-Whitney, Table 2). The median values for the individual specimens are reported in the S1 Fig. Although the magnitude of the moment in both direction of torsion was the same, the magnitude of the eps_1_ for clockwise and counterclockwise were different; conversely the eps_2_ were similar between clockwise and counterclockwise torsions. The eps_1_ were roughly oriented at +45° for clockwise torsion and -45° for counterclockwise torsion both on the vertebrae and intervertebral discs.

The plot of the strain in the circumferential direction of the ALL showed a pattern mirrored with respect to the vertical axis for clockwise and counterclockwise torsions (Fig 7). No differences were present in terms on magnitude between the two axial torsion scenarios. The distribution of strains in the cranio-caudal direction of the ALL in front of the vertebra was significantly different between clockwise and counterclockwise torsion only for eps_2_; conversely, the distributions in the discs were significantly different in all cases (two-sample Kolmogorov-Smirnov, Table 3).

### Specimen-specific analysis

The specific findings for the individual specimens are reported in terms of strain distribution (Fig 8), also in relation to the peculiar bony-defects (e.g.: osteophytes, scoliosis, etc) of each specimen (Table 1):

- Specimen #1: this specimen was considered healthy based only on x-ray imaging, the CT scan was not available. The strain patterns showed a right/left symmetry for both flexion and extension, and a mirrored distribution of strains for right vs. left lateral bending, and for clockwise vs. counterclockwise torsion.
- Specimen #2: moderate osteophytes were visible at the both endplates of L4 and on the cranial endplate of L5. The L4-L5 segment had a score for the osteophyte formation of 11 points, equivalent to Grade 2 according to [27]. The DIC analysis highlighted some local intensification of the strain distribution in the ALL in front of the L4-L5 IVD, in correspondence of these osteophytes for all loading scenarios with exception of the left bending. Furthermore, a local thickening of the anterior wall of the vertebral body of L4 was visible in the CT scan. This corresponded to a region with lower strains in front of L4.
- Specimen #3: the CT images exhibited moderate degenerative signs, with prominent osteophytes on the cranial endplates of L4 and of L5 (i.e.: one on the right, one on the left of each vertebra), which could act as strain concentrators cranially, while shielding the strains in the ALL surrounding them. The L4-L5 segment had a score for the osteophyte formation of 11 points, equivalent to a Grade 2. The DIC analysis showed an intensification of the strains in front of L3-L4 and L4-L5 IVDs, in the most cranial portion of the disc, and areas with much lower strains caudal to these spots.
- Specimen #4: this specimen was mildly scoliotic with a Cobb angle T12-L5 of 10° and concavity on the right side. The DIC analysis showed a non-mirrored strain pattern between right and left lateral bending, and between clockwise and counterclockwise torsion. Furthermore, it had several osteophytes at all endplates. The L4-L5 segment had a score for the osteophyte formation of 15 points, equivalent to a Grade 2. The DIC analysis revealed strain concentrations near such osteophytes. The largest osteophyte projected upwards from the central-left margin of the superior endplate of L5 partially covering the caudal portion of the L4-L5 IVD. In this area a strain attenuation was visible in the area where the osteophyte covered the IVD, especially for flexion.
- Specimen #5: the CT images showed that this specimen had prominent osteophytes on the right and left sides of the cranial endplates of L5. The L4-L5 segment had a score for the osteophyte formation of 17 points, equivalent to a Grade 3. The strain distributions showed an intensification in the right and left areas circumferentially the L4-L5 IVD in flexion.

More details about the individual specimens, the distribution of strains of the vertebrae and IVD in the circumferential and cranio-caudal directions can be found in the S1 Fig, S1 and S2 Dataset.

## Discussion

The aim of this work was to explore the biomechanical behaviour of the most superficial layer of the anterior longitudinal ligament focusing on the anterior aspect of the lumbar vertebrae and intervertebral disc, applying a new paradigm. Structural flexibility tests and local strain analysis were performed on the anterior surface of spine segments loaded in flexion/extension, lateral bending and torsion. The hypotheses of the work were that: (i) the strain field on the surface of the ALL is not homogeneous between different regions (i.e.: in front of the vertebrae and of the intervertebral discs); (ii) the strain distribution is not homogenous within each such region, possibly due to specific bony-defects; (iii) inside the same region the strain field depends on the different loading scenarios; and (iv) opposite directions of loading translate to non-mirrored strain distributions.

In order to test these hypotheses, segments of multi-vertebra human spine segments, to reproduce a better loading transmission on the ALL, were used. Each specimen was tested in flexion/extension, lateral bending and axial torsion up to 7.5 Nm with a state-of-the-art spine tester [29]. The global ranges of motion and neutral zones were identified for each specimen and each loading scenario through flexibility tests using optical motion tracking system. These data were integrated with a full-field measurement of the strain distribution in front of 3 lumbar vertebrae and intervertebral discs using a validated digital image correlation approach [25,34].

The global range of motion under load and the evaluation of the neutral zone, for the different loading scenarios, confirmed the typical trend and values for human lumbar spine, as reported in the Busscher et al. work [10,35]. They showed that segments from L1 to L4 at 4 Nm had a range of motion of 5° in flexion/extension, 6° in lateral bending and 2° in axial torsion. These results were in accordance with our study, indeed for L1-L4 segments at 4 Nm the following range of motion were obtained: 5.6° flexion/extension, 7.8° lateral bending and 2.9° axial torsion. The full-field strain maps (Figs 2, 4 and 6 and S1 Fig) highlighted the non-homogeneity of strain in the different areas of the ALL: different trends were observed both in the cranio-caudal and circumferential direction of the ALL for the different loading conditions (Figs 3, 5 and 7 and S1 Fig). The strain fields suggested that some fibers were pronouncedly more strained than the rest of the ALL during loading, both in front of the vertebra and of the IVDs. Furthermore, there was a clear effect of the stress concentrators: in most specimens, also some spots with larger strains were visible, especially close to the endplates. No strain concentration was detected close to the markers screws insertions, confirming that the ALL was not damaged during the flexibility test. A detailed inspection of the CT scans of the specimens highlighted that such strain concentrations corresponded to the position of local osteophytes and bony-defects (See Fig 8 and the S1 Fig for details). For instance, protruding osteophytes were associated with strain concentrations towards the tip of the osteophyte, but shielded the ALL in the areas where the osteophyte covered the IVD. Interestingly, despite osteophytes were found to reduce the flexibility of a severely degenerated spine [36,37], our results may contribute in elucidating the underlying biomechanical principles. A possible interpretation, suggested by the peculiar morphology of the osteophytes protruding from the endplates, may be that the relative distance of the outer ALL layers from to the instantaneous center of rotation of the FSU (i.e. lever arm) [38] is increased, thus, resulting in a higher local strain. A further characterization of the local tissue composition and properties would be needed in order to clarify this aspect.

The most strained portion of the ALL superficial layers was in front of the IVDs with a strain magnitude that was between 1.15 and 8.12 times larger than in front of the vertebrae (Figs 2, 4 and 6); these differences were statistically significant only for some loading scenarios (Table 2). This condition could be due to a series of reasons: the ALL in IVDs regions is thinner compared to the regions in front of the vertebrae [16], the ALL deep layers are less constrained in front of the IVD than in front of the vertebra [1], in front of the IVD the ALL is subjected to the large deformation of the IVD itself [23]. It is worth noting that, while the vertebral bone is at least two orders of magnitude stiffer than the adjacent IVD [39–41], the differences in strain of the superficial layer of the ALL in front of the vertebra and disc were relatively smaller: this is probably explained by the fact that the ALL act as a long ribbon, spanning across multiple FSU, with some motion relative to the underlying bone. Among the different direction of bending (flexion, extension, and lateral bending), flexion resulted in the highest strains and therefore seemed the most demanding loading scenario for the ligament (Fig 2). In fact, due to the action of the underlying pressurized and bulging discs, the ALL is largely strained circumferentially. During flexion, the ALL can provide only a limited direct contribution to the spinal stability [42]. However, the presence of large strains in the ALL seems to be due to anterior disc bulging during flexion, and indicates a role of the ALL in protecting the discs against herniation on the anterior face. Such a finding is consistent with [22], who observed that strain increased in the anterior portion of the IVD after ALL removal. Nevertheless, the ALL may have a significant bi-axial pre-strain *in vivo* depending on the region where it is attached (IVD or vertebra) [3,16]. As the only way to measure a pre-strain is through destructive testing, this phenomenon cannot be captured by our current non-destructive analysis.

Also in extension the ALL in front of the IVD and of L4 underwent an appreciable longitudinal strain, confirming the important mechanical role of ALL in constraining extension, in conjunction with the action of the facet joints [41].

Lateral bending seemed to be the loading scenario that strained less the ALL in terms of absolute values of strains. This is due because the ALL covers the regions in proximity of the neutral axis for lateral bending. Nevertheless, the strain distribution during right and left lateral bending was rather mirrored with respect to the vertical axis in front of the disc but not in front of the vertebra (Figs 4 and 5, and Table 3). While the strains in the circumferential direction of the ALL for the left lateral bending showed the trend that one would expect based on the distribution of tension/compression in bending, this trend was not confirmed for right lateral bending. It is possible to hypothesize that this systematic difference was due to the scoliosis of the donors. Unfortunately, no information was available about their dominant side (left-handed or right-handed) that could influence this systematic difference.

The torsional scenario was associated with large strains in the ALL, with smaller differences between the regions in front of the vertebrae and of the IVD (Fig 6). The lack of symmetry between right and left torsion (Fig 7 and Table 3) could again be explained by some asymmetry due to laterality of the donors. Furthermore, scoliotic specimens showed different strain maps between bending in the two directions, and between the two opposite directions of torsion. Therefore, not only we were able to identify general trends, but also to detect localized effect of large and small anatomical anomalies.

To the best authors’ knowledge, this is the first work where the full-field strain distributions were computed on the ALL in lumbar spine segments. Previous works explored the mechanical behaviour of ALL through strain analysis. [20,21] measured the mechanical properties of the ALL *in situ* under pure tension, after removal of the IVD. The evaluation of the tensile strain was performed on macro-regions of the ALL: insertions and free-length, in the cranio-caudal direction of the ALL; outer and central regions, circumferentially the ALL. They showed larger strain in the substance and outer regions of the ligaments, similar to the present study. [22,23,43] evaluated the strain on the entire surface of the intervertebral discs through a laser scanner device while the FSU segments was loaded in the same spine tester and the same conditions as the present work. The strain magnitude and distribution reported in those papers are comparable with the median strains obtained in the present work, confirming the suitability of the measurement technique and corroborating the present results.

Other works studied the mechanical properties of the ALL removing it from the spine and testing it in pure tensile tests. These conditions were far from the scenarios implemented in the present study; nevertheless some qualitative comparisons are possible. [16–18] revealed the weakness of the ALL at the level of the IVDs. Unfortunately, the non-destructive testing procedure did not allow to analyze the different layers of the ALL and how each layer influenced the strain distribution. According to our observations, we can surely appreciate that under consistent loading conditions, specific regions of different specimens lead to comparable strain patterns.

A limitation of the present work is the sample size: five specimens have a limited statistical power. It worth noticing that the results here reported are in agreement with the kinematics data reported for a larger specimens’ cohort [44]. As this study is extremely demanding in terms of costs, testing and strain analysis, it was not possible to extend to a larger sample. However, the differences between the different anatomical regions, and between loading scenarios were sufficiently large to show statistical significance in most cases.

Some specimens showed some typical defects of elderly donors, such as osteophytes and consequent disc degeneration. Our detailed DIC strain investigation on the region of interest allowed identifying the associated perturbations on the strain distributions. However, some of the results may be biased by a relatively high BMI of the donors, and a relatively low BMD. While the present findings are directly applicable to spines of subjects with similar BMI, an extension to cases with normal BMI is possible because the donors were physically active until death, and therefore their ALL can be expected to have normal mechanical properties. Conversely, slightly different behaviour would possibly be observed in healthier spines with different BMD: the bone/ligaments insertions would play a fundamental role, in particular their stiffness could modify the local behaviour of the ALL.

The experimental setup had intrinsic limitations such as the reduced loading rate, which is far from physiological [45]. This was necessary to ensure that the soft tissues were not subjected to trauma, and a series of cycles can be repeated [30]. Furthermore, the focus of this study was not on the absolute magnitude of the strains which would be affected by the loading rate, but on a comparison between different regions and different loading scenarios under quasi-static loading conditions. Although, muscles forces and weight contribution were not considered in the current study, pure unconstrained moments remains the preferred option for *in vitro* reproducing relevant loading conditions [28]. Furthermore, this loading condition allows better control and reproducibility compared to follower loads (an experimental technique for applying compressive loads along the whole spine segment) or a compressive load [46–48].

The accuracy and precision of the DIC was optimized for each acquisition; however, testing fresh specimens entailed leakages of biological fluid that can lead to some local loss of correlation [25]. In the worst case, correlation was lost on 20% of the region of interest. Nevertheless, the entire acquisition and post-processing protocol allowed to clearly show what happened in the different specimens and different loading conditions. Finally, only what happened on the visible surface of the ligaments was evaluated. Currently, it is the only possible compromise to study the ALL in physiological range of motion.

## Conclusions

This is the first time that the distribution of strain in the anterior longitudinal ligament was measured in multi-vertebra intact spine segments. The obtained results showed the non-uniform strain distribution, under the different loading scenarios. The vertebrae and intervertebral discs, with their peculiar defects (e.g.: osteophytes, etc.) played a fundamental role in defining the behaviour of the ALL. The current analysis including a spine tester and an unpreceded measurement of the strain distribution is so detailed that not only we could investigate the average effects of the different loading scenarios, but also the local effect that subject-specific defects may have on strain distribution. These results suggested again the importance of a full-field strain analysis to understand the biomechanics of the human spine and the interaction between different tissue types. This work could be the starting point for future studies where the effect of surgical procedures will be compared with intact spines.

## Supporting information

S1 Fig Specimens analysis. For each specimen (#1 to #5, in separate sheets) the following are reported:
• Left: An image of the specimen with an indication of the vertebrae and disc under consideration, and of the sub-ROIs where strains were computed along and around the ALL in front of the L4 vertebra and in front of the L4-L5 IVD.
• For each loading scenario, the maps of the maximum (eps_1_) and minimum (eps_2_) engineering principal strains are shown in the top images. Below each loading scenario, the distributions of the maximum (eps_1_) and minimum (eps_2_) strains are plotted around and along the ALL, both in front of the L4 vertebra and of the L4-L5 IVD.
• On the right, a volumetric reconstruction of the vertebrae from the CT scan is reported: the circles highlight the osteophytes, graded as 1 (<3 mm, yellow circle), 2 (between 3 and 6 mm, orange circle) or 3 (>6 mm, red circle) according to [27]
The table at the bottom right reports the median strains (maximum (eps_1_) and minimum (eps_2_) engineering principal strains) both in front of the L4 vertebra and in front of the L4-L5 IVD, for each loading scenario (flexion, extension, right bending, left bending, clockwise torsion, counterclockwise torsion).
(PDF)

S1 Dataset Raw data at max load. The maximum (eps1) and minimum (eps2) principal strains measured through the Digital Image Correlation on each specimen were reported with their spatial coordinates.
(XLSX)

S2 Dataset Markers for local analysis. The spatial coordinates of the regions of interest in front of the vertebra and the in front of the intervertebral disc were reported for each specimen for each loading condition in both the directions.
(XLSX)

## References

[1] Gray H. Gray’s anatomy: the Anatomical Basis of Medicine and Surgery. Churchill-Livingstone, 2004

[2] Mercer S, Bogduk N. The ligaments and annulus fibrosus of human adult cervical intervertebral discs. Spine. 1999 pp. 619–26; discussion 627–8. 10.1097/00007632-199904010-00002 10209789

[3] Robertson DJ, Von Forell GA, Alsup J, Bowden AE. Thoracolumbar spinal ligaments exhibit negative and transverse pre-strain. J Mech Behav Biomed Mater. Elsevier; 2013;23: 44–52. 10.1016/j.jmbbm.2013.04.004 23660304

[4] Rhalmi S, Yahia L, Newman N, Isler M. Immunohistochemical study of nerves in lumbar spine ligaments. Spine (Phila Pa 1976). 1993;18: 264–7. 8441943 10.1097/00007632-199302000-00015

[5] Brandolini N, Cristofolini L, Viceconti M. Experimental Method for the Biomechanical Investigation of Human Spine: a Review. J Mech Med Biol. 2014;14: 1430002.

[6] Wilke H-J, Rohlmann A, Neller S, Schulthei M, Bergmann G, Graichen F, et al Is It Possible to Simulate Physiologic Loading Conditions by Applying Pure Moments?: A Comparison of In Vivo and In Vitro Load Components in an Internal Fixator. Spine (Phila Pa 1976). 2001;26: 636–642. 11246374 10.1097/00007632-200103150-00014

[7] Oxland TR. Fundamental biomechanics of the spine—What we have learned in the past 25 years and future directions. J Biomech. 2016;49: 817–832. 10.1016/j.jbiomech.2015.10.035 26706717

[8] Lin HS, Liu YK, Adams KH. Mechanical response of the lumbar intervertebral joint under physiological (complex) loading. J Bone Jt Surg Am. 1978;60: 41–55. 624758

[9] Kettler a, Wilke HJ, Haid C, Claes L. Effects of specimen length on the monosegmental motion behavior of the lumbar spine. Spine (Phila Pa 1976). 2000;25: 543–50. 10.1097/00007632-200003010-00003 10749629

[10] Busscher I, van Dieen JH, Kingma I, van der Veen AJ, Verkerke GJ, Veldhuizen AG. Biomechanical Characteristics of Different Regions of the Human Spine. Spine (Phila Pa 1976). 2009;34: 2858–2864. 20010393 10.1097/BRS.0b013e3181b4c75d

[11] Wilke HJ, Geppert J, Kienle A. Biomechanical in vitro evaluation of the complete porcine spine in comparison with data of the human spine. Eur Spine J. 2011;20: 1859–1868. 10.1007/s00586-011-1822-6 21674213 PMC3207338

[12] Wilke HJ, Krischak ST, Wenger KH, Claes LE. Load-displacement properties of the thoracolumbar calf spine: experimental results and comparison to known human data. Eur Spine J. 1997/01/01. 1997;6: 129–137. 10.1007/BF01358746 9209882 PMC3454592

[13] Panjabi MM, Oxland TR, Yamamoto I, Crisco JJ. Mechanical behavior of the human lumbar and lumbosacral spine as shown by three-dimensional load-displacement curves. J Bone Jt Surg Am. 1994;76: 413–424. 10.2106/00004623-199403000-00012 8126047

[14] Gardner-Morse MG, Stokes IAF. Structural behavior of human lumbar spinal motion segments. J Biomech. 2004;37: 205–212. 10.1016/j.jbiomech.2003.10.003 14706323

[15] Lysack JT, Dickey JP, Dumas GA, Yen D. A continuous pure moment loading apparatus for biomechanical testing of multi-segment spine specimens. J Biomech. 2000;33: 765–770. 10.1016/s0021-9290(00)00021-x 10807999

[16] Tkaczuk H. Tensile Properties of Human Lumbar Longitudinal Ligaments. Acta Orthop Scand. 1968;39: 1–69. 10.3109/17453676808989433 5707337

[17] Chazal J, Tanguy A, Bourges M, Gaurel G, Escande G, Guillot M, et al Biomechanical properties of spinal ligaments and a histological study of the supraspinal ligament in traction. J Biomech. 1985;18: 167–176. 10.1016/0021-9290(85)90202-7 3997901

[18] Pintar FA, Yoganandan N, Myers T, Elhagediab A, Sances JA. Biomechanical Properties of Human Lumbar Spine Ligaments. J Biomech. 1992;25: 1351–1356. 10.1016/0021-9290(92)90290-h 1400536

[19] Panjabi MM, Goel VK, Takata K. Physiologic Strains in the lumbar spinal ligaments. An In Vitro Biomechanical Study. 1982;7: 192–203. 10.1097/00007632-198205000-00003 6214027

[20] Neumann P, Ekström LA, Keller TS, Perry L, Hansson TH. Aging, vertebral density, and disc degeneration alter the tensile stress‐strain characteristics of the human anterior longitudinal ligament. J Orthop Res. 1994;12: 103–112. 10.1002/jor.1100120113 8113932

[21] Neumann P, Keller TS, Ekstrom L, Perry L, Hansson TH, Spengler DM. Mechanical Properties of the Human Lumbar Anterior Longitudinal Ligament. J Biomech. 1992;25: 1185–1194. 10.1016/0021-9290(92)90074-b 1400518

[22] Heuer F, Schmidt H, Wilke HJ. Stepwise reduction of functional spinal structures increase disc bulge and surface strains. J Biomech. 2008;41: 1953–1960. 10.1016/j.jbiomech.2008.03.023 18501361

[23] Heuer F, Schmidt H, Wilke HJ. The relation between intervertebral disc bulging and annular fiber associated strains for simple and complex loading. J Biomech. 2008;41: 1086–1094. 10.1016/j.jbiomech.2007.11.019 18187139

[24] Palanca M, Tozzi G, Cristofolini L. The Use Of Digital Image Correlation In The BIomechanical Field: A Review. Inter Biomech. 2016; 3: 1–21. 10.1080/23335432.2015.1117395

[25] Palanca M, Marco M, Ruspi ML, Cristofolini L. Full-field strain distribution in multi-vertebra spine segments: an in-vitro application of DIC. Med Eng Phys. 2018;52: 76–83. 10.1016/j.medengphy.2017.11.003 29229402

[26] Palanca M, Barbanti-Bròdano G, Cristofolini L. The Size of Simulated Lytic Metastases Affects the Strain Distribution on the Anterior Surface of the Vertebra. J Biomech Eng. 2018;140: 111005 10.1115/1.4040587 30029268

[27] Wilke HJ, Rohlmann F, Neidlinger-Wilke C, Werner K, Claes L, Kettler A. Validity and interobserver agreement of a new radiographic grading system for intervertebral disc degeneration: Part I. Lumbar spine. Eur Spine J. 2006;15: 720–730. 10.1007/s00586-005-1029-9 16328226 PMC3489460

[28] Wilke HJ, Wenger K, Claes L. Testing criteria for spinal implants: recommentations for the standardardization of in vitro stability testing of spinal implants. Eur Spine J. 1998;7: 148–154. 10.1007/s005860050045 9629939 PMC3611233

[29] Wilke HJ, Claes L, Schmitt H, Wolf S. A universal spine tester for in vitro experiments with muscle force simulation. Eur Spine J. 1994;3: 91–97. 10.1007/bf02221446 7874556

[30] Wilke H-J, Jungkunz B, Wenger K, Claes LE. Spinal segment range of motion as a function of in vitro test conditions: effects of exposure period, accumulated cycles, angular-deformation rate, and moisture condition. Anat Rec. 1998;251: 15–19. 10.1002/(SICI)1097-0185(199805)251:1<15::AID-AR4>3.0.CO;2-D 9605215

[31] Sutton MA, Orteu JJ, Schreier HW. Image Correlation for Shape, Motion and Deformation Meaasurements. Springer Sci. 2009;

[32] Lionello G, Cristofolini L. A practical approach to optimizing the preparation of speckle patterns for digital-image correlation. Meas Sci Technol. 2014;25: 107001 10.1088/0957-0233/25/10/107001

[33] Palanca M, Brugo TMM, Cristofolini L. Use of Digital Image Correlation to Understand the Biomechanics of the Vertebra. J Mech Med Biol. 2015;15: 1540004–1540010. 10.1142/S0219519415400047

[34] Palanca M, Tozzi G, Cristofolini L. The use of digital image correlation in the biomechanical area: A review. Int Biomech. 2016;3: 1–21. 10.1080/23335432.2015.1117395

[35] Wilke H-J, Kettler A, Claes L E. Are sheep spines a valid biomechanical model for human spines? Spine 1997; 22 (20): 2365–2374 10.1097/00007632-199710150-00009 9355217

[36] Tanaka N, An HS, Lim TH, Fujiwara A, Jeon CH, Haughton VM. The relationship between disc degeneration and flexibility of the lumbar spine. Spine J. 2001;1: 47–56. 10.1016/s1529-9430(01)00006-7 14588368

[37] Al-Rawahi M, Luo J, Pollintine P, Dolan P, Adams MA. Mechanical Function of Vertebral Body Osteophytes, as Revealed by Experiments on Cadaveric Spines. Spine (Phila Pa 1976). 2010; 30. 10.1097/BRS.0b013e3181df1a70 20683388

[38] Heuer F, Schmidt H, Claes L, Wilke HJ. Stepwise reduction of functional spinal structures increase vertebral translation and intradiscal pressure. J Biomech. 2007;40: 795–803. 10.1016/j.jbiomech.2006.03.016 16712856

[39] Costi JJ, Hearn TC, Fazzalari NL. The effect of hydration on the stiffness of intervertebral discs in an ovine model. Clin Biomech. 2002;17: 446–455. 10.1016/S0268-0033(02)00035-9 12135546

[40] Dall’Ara E, Schmidt R, Pahr D, Varga P, Chevalier Y, Patsch J, et al A nonlinear finite element model validation study based on a novel experimental technique for inducing anterior wedge-shape fractures in human vertebral bodies in vitro. J Biomech. Elsevier; 2010;43: 2374–2380. 10.1016/j.jbiomech.2010.04.023 20462582

[41] White III AA, Panjabi MM. Clinical Biomechanics of the Spine. Second Edi Lippincott Williams & Wilkins; 1990.

[42] Schmidt R, Obertacke U, Nothwang J, Ulrich C, Nowicki J, Reichel H, et al The impact of implantation technique on frontal and sagittal alignment in total lumbar disc replacement: A comparison of anterior versus oblique implantation. Eur Spine J. 2010;19: 1534–1539. 10.1007/s00586-010-1432-8 20490873 PMC2989294

[43] Heuer F, Wolfram U, Schmidt H, Wilke H-J. A method to obtain surface strains of soft tissues using a laser scanning device. J Biomech. 2008;41: 2402–2410. 10.1016/j.jbiomech.2008.05.031 18621375

[44] La Barbera L, Brayda-Bruno M, Liebsch C, Villa T, Luca A, Galbusera F, et al Biomechanical advantages of supplemental accessory and satellite rods with and without interbody cages implantation for the stabilization of pedicle subtraction osteotomy. Eur Spine J. 2018;27: 2357–2366. 10.1007/s00586-018-5623-z 29740675

[45] Race A, Broom ND, Robertson P. Effect of loading rate and hydratation on the mechanical properties of the disc. Spine (Phila Pa 1976). 2000;25: 662–669. 10752096 10.1097/00007632-200003150-00003

[46] Volkheimer D, Malakoutian M, Oxland TR, Wilke HJ. Limitations of current in vitro test protocols for investigation of instrumented adjacent segment biomechanics: critical analysis of the literature. Eur Spine J. 2015;24: 1882–1892. 10.1007/s00586-015-4040-9 26038156

[47] Rohlmann A, Neller S, Claes L, Bergmann G, Wilke HJ. Influence of a follower load on intradiscal pressure and intersegmental rotation of the lumbar spine. Spine (Phila Pa 1976). 2001/12/12. 2001;26: E557—61. 11740371 10.1097/00007632-200112150-00014

[48] Sis HL, Mannen EM, Wong BM, Cadel ES, Bouxsein ML, Anderson DE, et al Effect of follower load on motion and stiffness of the human thoracic spine with intact rib cage. J Biomech. 2016;49: 3252–3259. 10.1016/j.jbiomech.2016.08.003 27545081 PMC5702885

