# Supplementary material for: The strain distribution in the lumbar anterior longitudinal ligament is affected by the loading condition and bony features: An in vitro full-field analysis
Source: PLoS One. 2020 Jan 14;15(1):e0227210. doi: 10.1371/journal.pone.0227210 (PMC6959510; doi:10.1371/journal.pone.0227210)
Supplement: S1 Fig — For each specimen (#1 to #5, in separate sheets) the following are reported: • Left: An image of the specimen with an indication of the vertebrae and disc under consideration, and of the sub-ROIs where strains were computed along and around the ALL in front of the L4 vertebra and in front of the L4-L5 IVD. • For each loading scenario, the maps of the maximum (eps1) and minimum (eps2) engineering principal strains are shown in the top images. Below each loading scenario, the distributions of the maximum (eps1) and minimum (eps2) strains are plotted around and along the ALL, both in front of the L4 vertebra and of the L4-L5 IVD. • On the right, a volumetric reconstruction of the vertebrae from the CT scan is reported: the circles highlight the osteophytes, graded as 1 (<3 mm, yellow circle), 2 (between 3 and 6 mm, orange circle) or 3 (>6 mm, red circle) according to [27] The table at the bottom right reports the median strains (maximum (eps1) and minimum (eps2) engineering principal strains) both in front of the L4 vertebra and in front of the L4-L5 IVD, for each loading scenario (flexion, extension, right bending, left bending, clockwise torsion, counterclockwise torsion). (PDF) [file pone.0227210.s001.pdf]

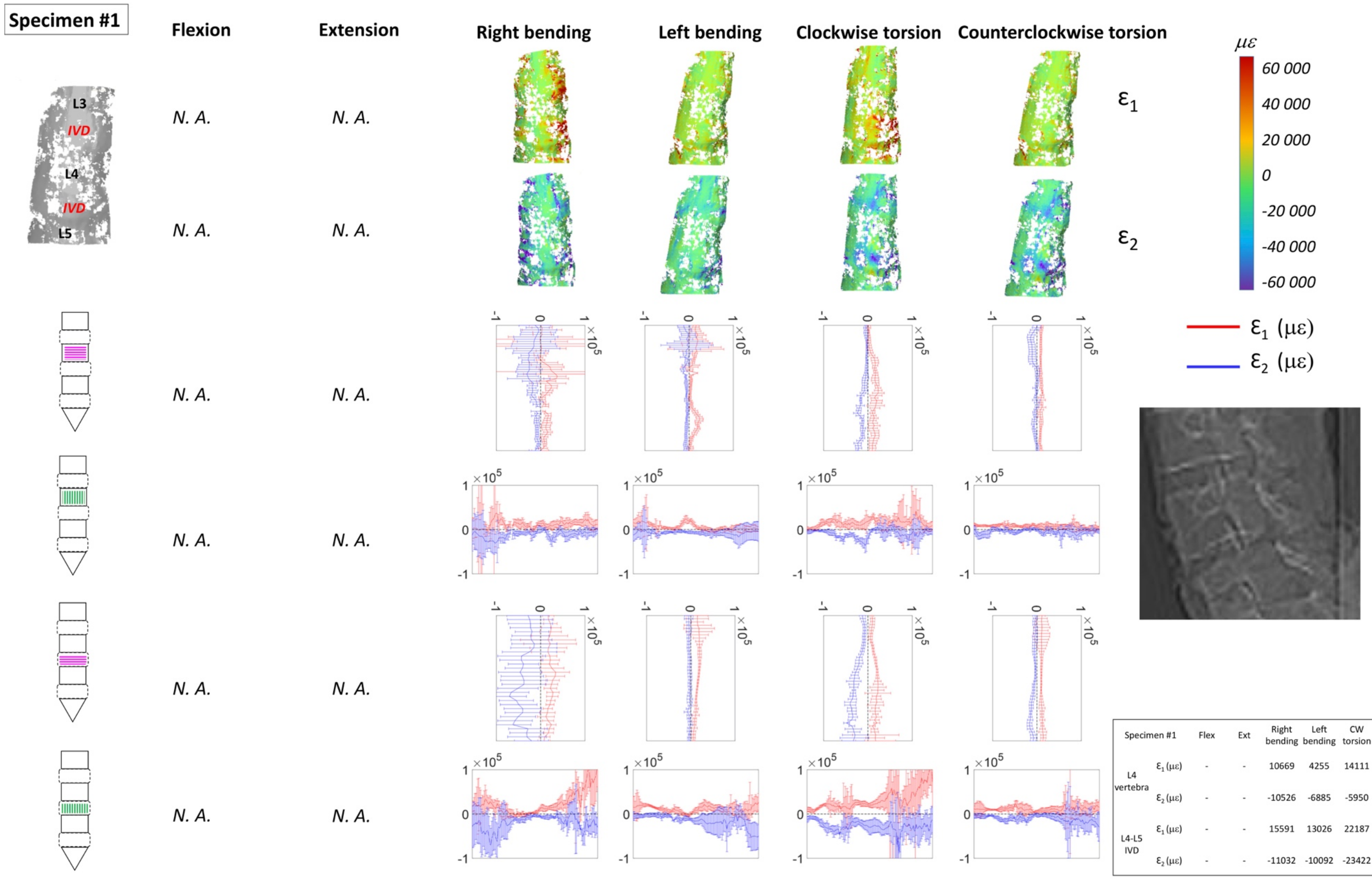

Specimen #1		Flex	Ext	Right bending	Left bending	CW torsion	CCW torsion
L4 vertebra	ε_1 ($\mu\varepsilon$)	-	-	10669	4255	14111	7611
	ε_2 ($\mu\varepsilon$)	-	-	-10526	-6885	-5950	-3691
L4-L5 IVD	ε_1 ($\mu\varepsilon$)	-	-	15591	13026	22187	10040
	ε_2 ($\mu\varepsilon$)	-	-	-11032	-10092	-23422	-7232

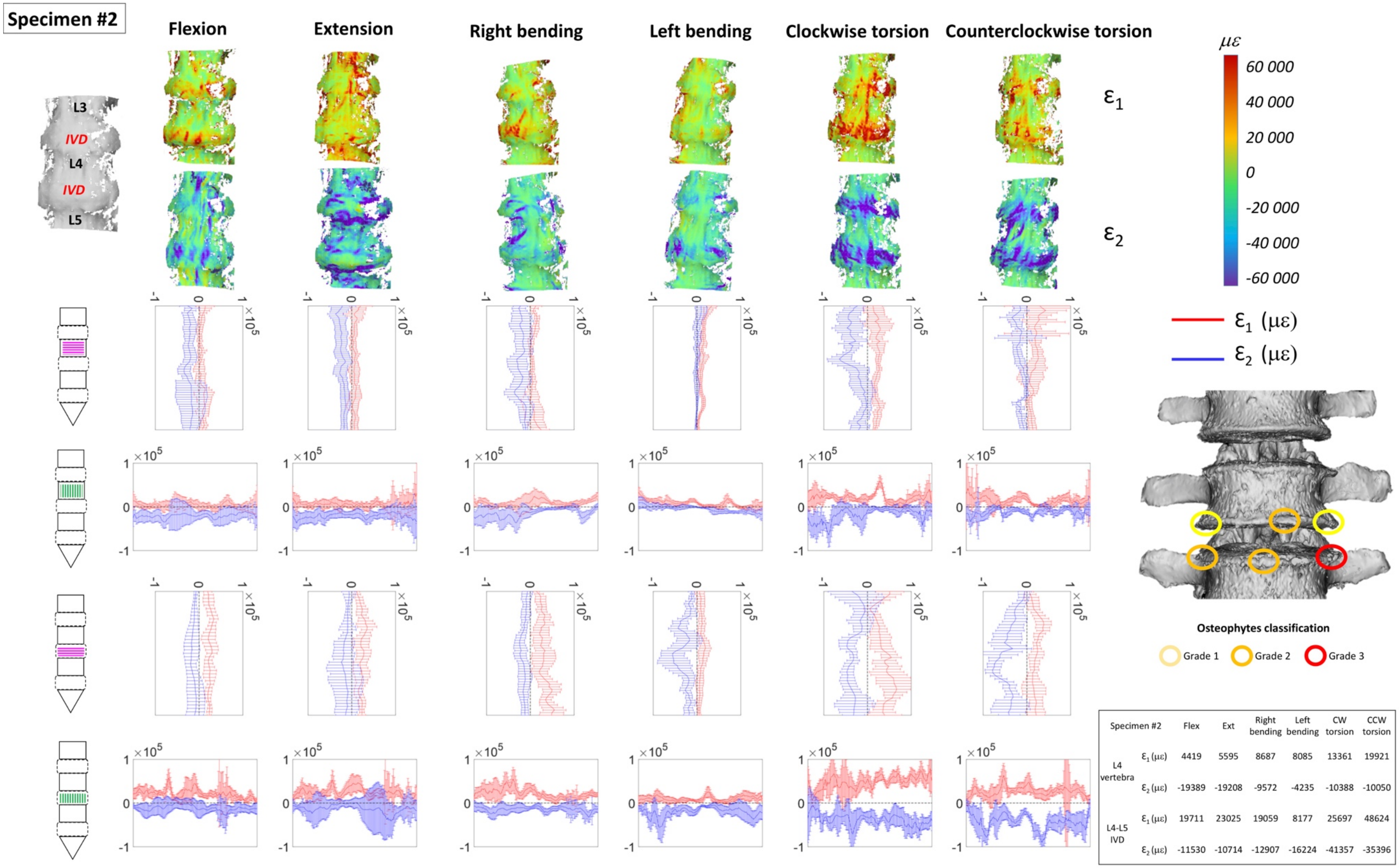

Specimen #2		Flex	Ext	Right bending	Left bending	CW torsion	CCW torsion
L4 vertebra	ε_1 ($\mu\varepsilon$)	4419	5595	8687	8085	13361	19921
	ε_2 ($\mu\varepsilon$)	-19389	-19208	-9572	-4235	-10388	-10050
L4-L5 IVD	ε_1 ($\mu\varepsilon$)	19711	23025	19059	8177	25697	48624
	ε_2 ($\mu\varepsilon$)	-11530	-10714	-12907	-16224	-41357	-35396

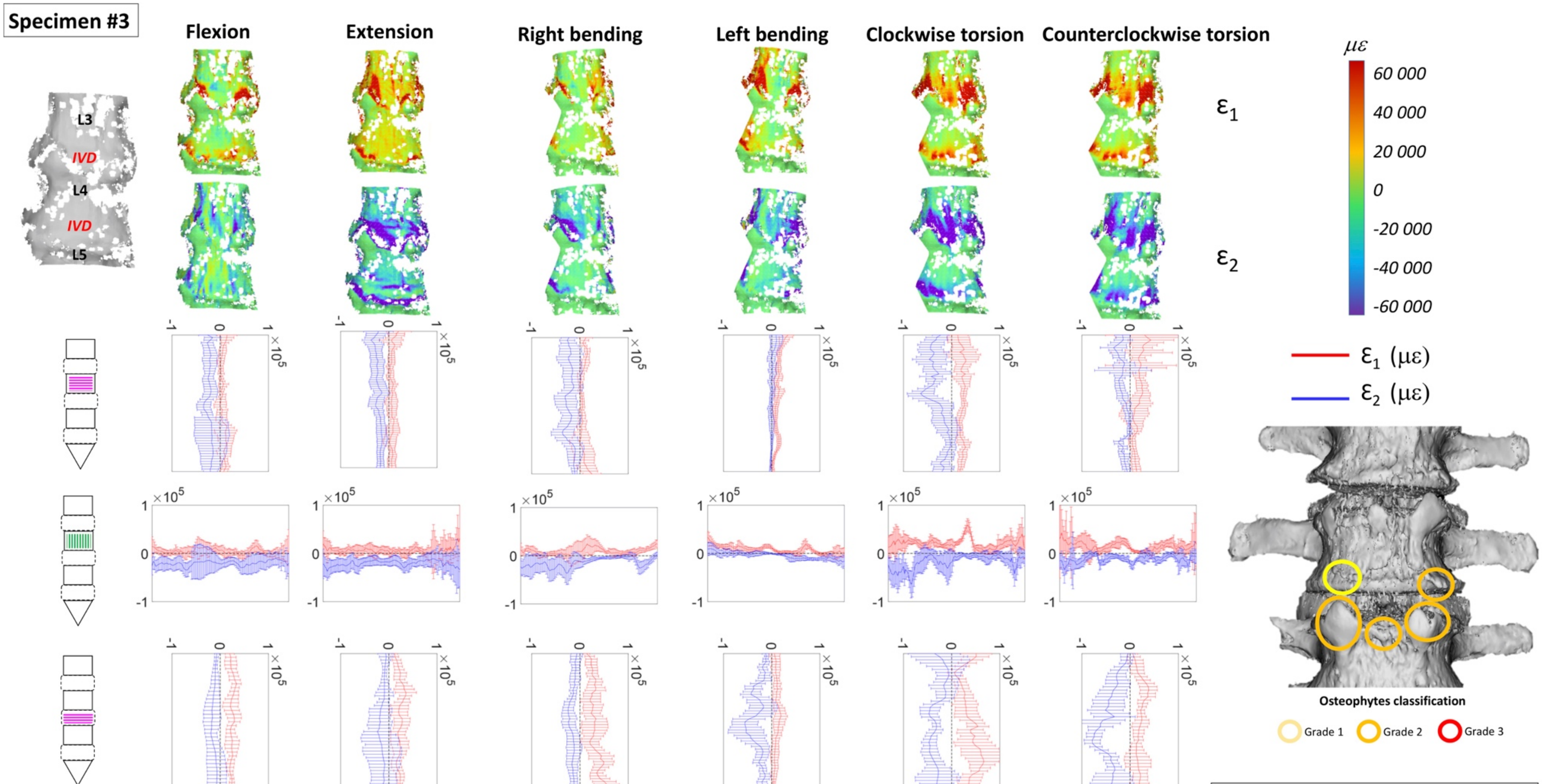

Specimen #3		Flex	Ext	Right bending	Left bending	CW torsion	CCW torsion
L4 vertebra	ε_1 ($\mu\varepsilon$)	3404	17996	3090	2695	3592	6446
	ε_2 ($\mu\varepsilon$)	-15321	-1892	-3679	-7371	-2244	-3859
L4-L5 IVD	ε_1 ($\mu\varepsilon$)	12948	18730	3557	8513	23169	38877
	ε_2 ($\mu\varepsilon$)	-23264	-5923	-4312	-5827	-21805	-31341

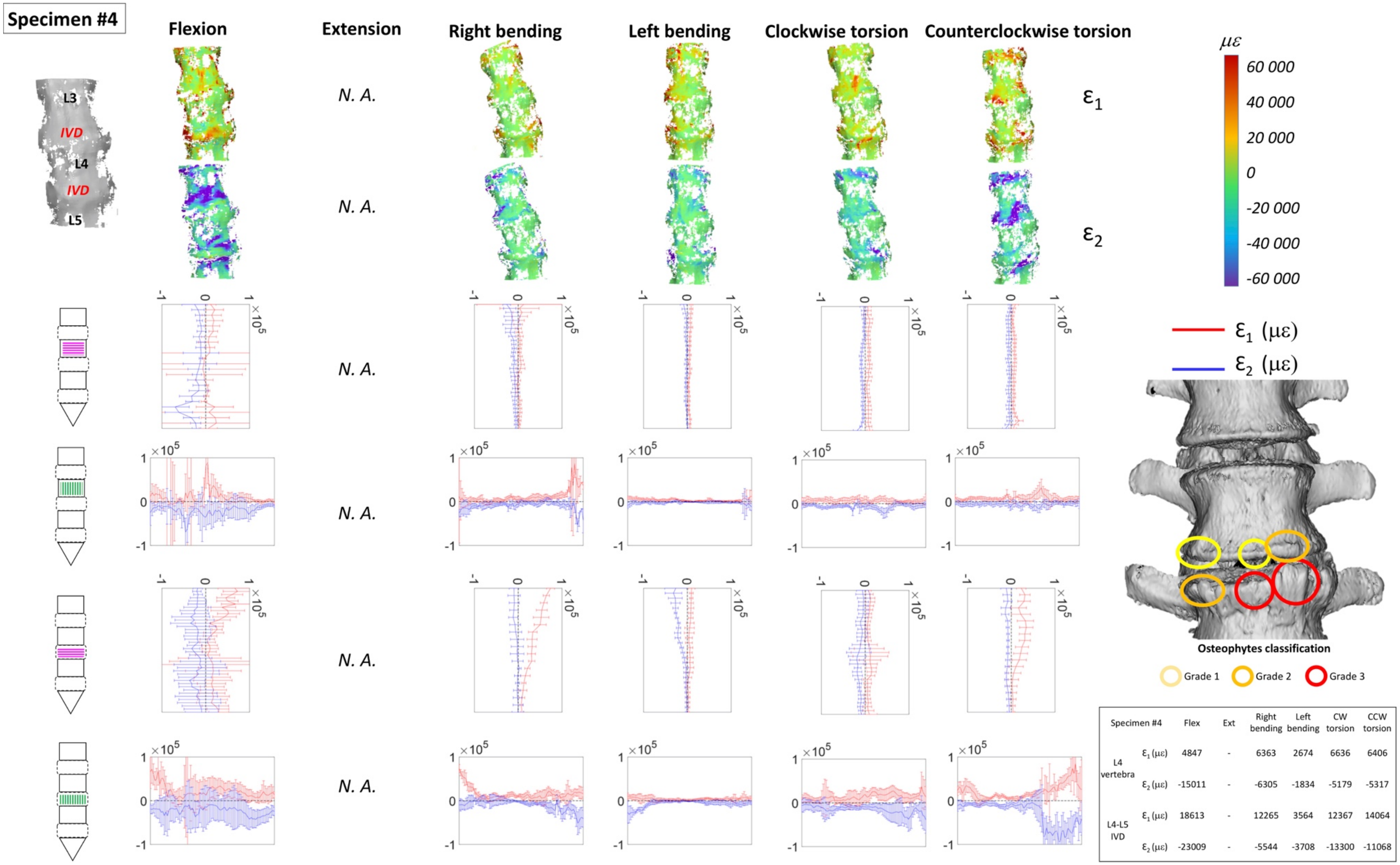

Specimen #4		Flex	Ext	Right bending	Left bending	CW torsion	CCW torsion
L4 vertebra	ε_1 ($\mu\varepsilon$)	4847	-	6363	2674	6636	6406
	ε_2 ($\mu\varepsilon$)	-15011	-	-6305	-1834	-5179	-5317
L4-L5 IVD	ε_1 ($\mu\varepsilon$)	18613	-	12265	3564	12367	14064
	ε_2 ($\mu\varepsilon$)	-23009	-	-5544	-3708	-13300	-11068

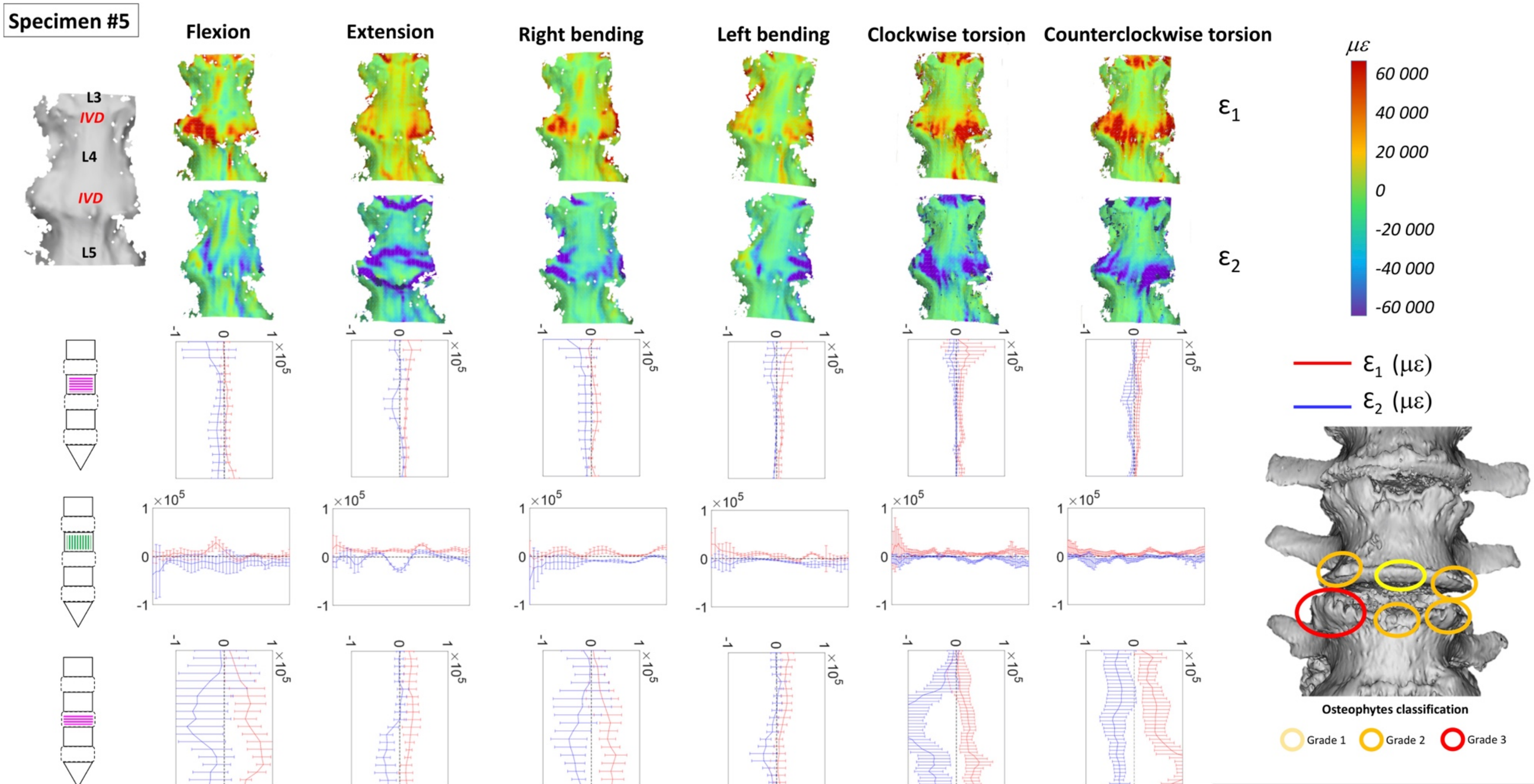

Specimen #5		Flex	Ext	Right bending	Left bending	CW torsion	CCW torsion
L4 vertebra	ε_1 ($\mu\varepsilon$)	2040	13892	5804	6454	7725	7352
	ε_2 ($\mu\varepsilon$)	-12442	-1685	-10071	-6598	-3549	-3070
L4-L5 IVD	ε_1 ($\mu\varepsilon$)	32690	18500	17271	11075	30553	41152
	ε_2 ($\mu\varepsilon$)	-23024	-12412	-19114	-12296	-30956	-36961
